# A manifesto for Alzheimer’s disease drug discovery in the era of disease-modifying therapies

**DOI:** 10.1186/s13024-025-00872-7

**Published:** 2025-08-06

**Authors:** Heike Hering, Thierry Bussiere, Chia-Chen Liu, Kelly E. Glajch, Andreas Weihofen, Jane Grogan, Dominic M. Walsh

**Affiliations:** 1 Neurodegeneration Research Unit, Biogen, Cambridge, MA 02142 USA; 2 Ann Romney Center for Neurologic Diseases, Brigham and Women’s Hospital and Harvard Medical School, Hale Building for Transformative Medicine, 60 Fenwood Road, Boston, MA 02115 USA

**Keywords:** Aggregation inhibitors, ⍺-synuclein, Amyloid β-protein, Antisense oligonucleotides, Apolipoprotein E, Brain shuttles, Gene therapy, Immunotherapy, Tau, Protein degraders, Small interfering RNA

## Abstract

After decades of disappointment, three disease-modifying therapies for Alzheimer’s disease (AD) have been approved since 2021. Burgeoning clinical data on these amyloid β-protein (Aβ) targeting drugs validate the amyloid cascade hypothesis as a molecular roadmap for the development of yet more effective therapeutics and offer a template for drugging other AD-associated aggregation-prone proteins. While there remains much to be learned about the molecular pathology of AD, the current state of knowledge is sufficient to expedite the delivery of new drugs. Mindful of the urgent need of patients, we recommend prioritizing efforts in four directions: finishing the job on Aβ, accelerating and diversifying efforts on tau, and expanding discovery on apolipoprotein E and ⍺-synuclein. For each target, we explain the scientific premise, current efforts, and possible new approaches. In the short- and medium-term, we advocate focusing on the technical innovations required to better drug these already well validated targets. While the focus of this review is on expediating development of monotherapies, the subsequent approval of such agents will enable add-on or combination approaches best suited to individual patients.

## Introduction

**Fig. 1:**
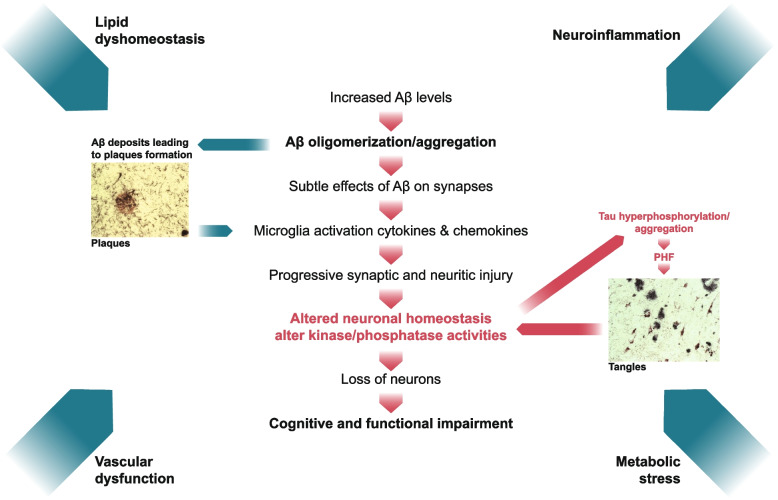
The amyloid cascade a molecular roadmap for understanding and drugging Alzheimer’s disease. Alzheimer’s disease is now widely considered as an Aβ driven tauopathy in which altered Aβ homeostasis is the common first step. Autosomal dominant forms of the disease are caused by mutations which change either the amount or the forms of Aβ produced, and certain risk factors alter the degradation, accumulation or response to Aβ. Burgeoning evidence indicates that Aβ directly influences tau metabolism and that tau can act both as a facilitator of Aβ-mediate toxicity and as a driver of neurodegeneration. The cascade is depicted as a linear series of reactions, but it is likely that certain steps occur simultaneously, and that multiple extrinsic factors act in a context dependent fashion to amplify or attenuate pathogenesis. Most GWAS-implicated AD risk factors link to neuroinflammation, lipid dyshomeostasis, vascular dysfunction and metabolic stress and each of these independently or in combination influence the molecular pathways driving an individual’s disease. Comorbidities and co-pathologies prevalent in older humans exacerbate and/or synergize with AD processes

Alzheimer’s disease (AD) is a complex syndrome defined by two pathological hallmarks: extracellular amyloid plaques primarily composed of the amyloid β-protein (Aβ), and intracellular neurofibrillary tangles of aggregated and hyperphosphorylated tau [1–4]. Extensive genetic, natural history, biomarker, molecular modeling, and recent clinical trial data indicate that Aβ plays a central and initiating role in AD [4–9]. It is not yet fully understood how changes in Aβ homeostasis drive disease, but it is widely believed to involve synaptic and neuronal dysfunction, activation of microglia, and ultimately synaptic and neuronal loss (Fig. 1).

While strong genetic and epidemiological data support alterations in lipid homeostasis, neurovascular health, and innate immunity [10–12], it is uncertain how these factors interact with the Aβ-tau axis to effect pathogenesis. Despite this incomplete picture, it is self-evident that the molecular sequelae that cause AD are not identical for all patients. Nonetheless, certain primary drivers are likely to be common, with disease manifesting via different routes depending on interplay with specific modulating factors and the presence of comorbidities and pathologies. Consistent with the importance of a common core of pathologies, the field has been quick to embrace the biological definition of AD as a continuum first recognized by changes in Aβ in asymptomatic individuals which then progress through stages of increasing pathologic burden and worsening clinical symptoms [4].

Initially, the US Food and Drug Administration and separately the National Institute on Aging-Alzheimer's Association working group proposed six stages based on detection of changes in amyloid (A), tau (T), and neurodegeneration (N) that can be monitored in life [13, 14]. More recently, these criteria were elaborated to include a Stage 0 for individuals free of pathology, but genetically predetermined to develop AD, and to incorporate emerging biomarkers for inflammation (I), vascular injury (V), and ⍺-synuclein (⍺Syn) pathology [4]. These frameworks recognize that AD pathology is not uniform across the brain but evolve in rather specific spatiotemporal patterns [15–18]. This means that at any instance, different regions of the same brain are experiencing different stages of pathogenesis. Consequently, it is likely that truly efficacious treatment of symptomatic AD (Stage 3 and beyond) will require the use of two or more agents. For the purpose of this Review, we address only monotherapy approaches, but expect that in the future, successful monotherapies will be tested in combinations.

We believe that the current understanding of AD pathobiology is sufficient to expedite development of new drugs, and that the primary challenges for the field in the short- and medium-term do not require identification of novel targets, but technical advances to better drug already well validated targets. Focus on the four recommended targets and the implementation of an intervention strategy applicable to all aggregation-prone proteins allows an economy of scale to pursue multiple modalities for each. This approach should realize the long-term goals of maximizing efficacy, safety and patient convenience, minimizing cost to payers, and treating the maximum number of people as early in the disease as possible.

## Building on success to maximize the efficacy of targeting Aβ

Clinical trials of antibodies that preferentially recognize aggregated Aβ (aducanumab, lecanemab, and donanemab) have shown to reduce in parenchymal amyloid burden and slow functional deterioration [8, 9, 19]. While the effect of these drugs on disease progression is modest, they are nonetheless real and critical for the patients they serve. Equally important, they provide a foothold on which to make progress to yet more efficacious Aβ-targeting therapies. Here, we will review the basics of Aβ production and aggregation, and how this information can be used to develop the next generation of anti-Aβ therapeutics, and in subsequent sections, we will discuss how this approach may be applied to the other aggregation-prone proteins involved in AD.

Aβ is a normal physiological product, and it has been estimated that a healthy individual produces somewhere between 1–3 kg of Aβ over the course of a 70 year life span [20]. However, Aβ is not a single molecular entity, but a family of closely related molecules that share a common core with considerable N- and C-terminal heterogeneity, and may undergo certain post-translational modifications [21]. The most common Aβ alloform in biological fluids is 40 residues long, but Aβ can extend up to 43 residues, and increased production of C-terminally extended forms of Aβ, particularly (but not exclusively) Aβ42, is associated with disease [22, 23]. Aβ is produced from the sequential enzymatic cleavage of the type-1 transmembrane amyloid precursor protein (APP). Initial cleavage by β-secretase (β-amyloid cleaving enzyme—BACE) leads to shedding of a large portion of the ectodomain (sAPPβ) and generation of a membrane-bound C-terminal fragment (CTFβ). Progressive processing of CTFβ by γ-secretase releases Aβ species with varying C-termini, including Aβ43, Aβ42, Aβ40, Aβ39/38, and Aβ37 [24]. Irrespective of its length, the Aβ monomer has a short half-life and is rapidly cleared under normal conditions; however, at locally high concentrations, Aβ can self-associate [25]. Evidence from in vitro and ex vivo experiments suggests that Aβ aggregation involves both primary and secondary nucleation producing a variety of different assemblies [26], some of which are on pathway to fibrils and some which are not. While fibrils have high thermodynamic stability, they and other Aβ aggregates can dissociate under certain circumstances. Large macroscopic aggregates of Aβ constitute amyloid plaques, diffuse amyloid, and cerebral amyloid angiopathy (CAA). What form or forms of Aβ cause AD remains unclear; however, there is widespread agreement that monomeric Aβ is innocuous, that aggregation is essential for pathogenesis, and removal of positron emission tomography (PET)-detectable amyloid (whether a biomarker or a mediator of disease) has therapeutic benefit.

**Fig. 2:**
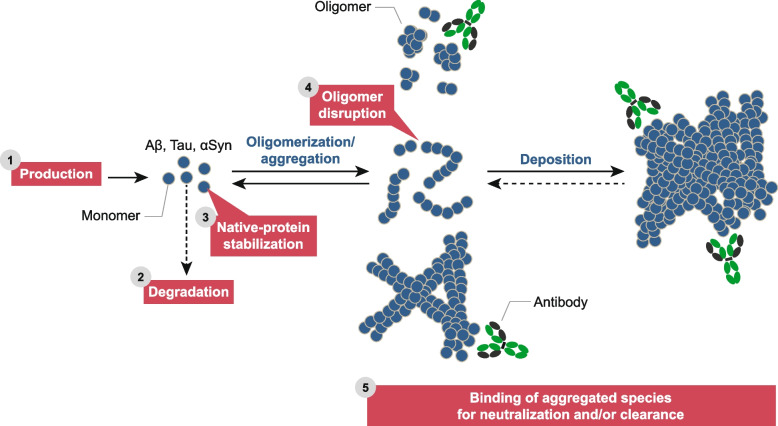
Shared strategies for drugging aggregation-prone proteins involved in AD. Aggregation of proteins is a concentration dependent process such that factors which control the concentration of unaggregated protein monomers can be exploited to prevent aggregation and potentially remove already formed aggregates. Reducing monomer levels (1) can be achieved at the DNA, RNA or protein level, and such approaches are relevant to both intracellular and extracellular targets. Facilitating degradation of monomers (2) should have the same outcome as inhibiting monomer production, however, the approaches to achieve enhanced degradation are necessarily different for intracellular and secreted proteins. Agents that bind to and stabilize the native protein (3) should allow for the natural removal of the protein by the brain’s degradative machinery, however, given that Aβ, tau and αSyn* are natively unfolded it is challenging to identify agents that stabilize what are inherently dynamic structures. Agents capable of disrupting aggregation intermediates (aka oligomers) (4) should reduce the concentration of oligomers and if these are on-path to fibrils may prevent fibrillogenesis and the deposition of larger aggregates. Antibodies or small molecules capable of binding to various abnormal assemblies (5) could neutralize the activity of oligomers and depending on their isotype facilitate the clearance of deposited aggregates by phagocytic myeloid cells. However, the ability to neutralize and/or remove aggregates will be governed by whether agents can access their target. Aβ aggregates are largely extracellular and antibodies with effector function readily mediate their removal. On the other hand, the predominant aggregates of tau and αSyn are intracellular and therefore largely inaccessible to antibodies and microglial mediated mechanisms for removal. However, should the formation of NFTs and Lewy bodies require the movement of aggregates from one neuron to another, then antibodies may be able to prevent further spreading. Antibodies without effector function could act to directly neutralize seeds, while antibodies with effector function could neutralize their target and facilitate its removal by immune cells. *Evidence suggests that α-synuclein may exist as a tetramer and therefore could be stabilized with an appropriate small molecule.

Theoretically, there are at least five ways to intervene therapeutically in the amyloid aggregation pathway (Fig. 2): (1) prevent production of Aβ monomer, (2) enhance degradations of Aβ monomer and/or aggregates, (3) stabilize monomer to prevent aggregation, (4) disaggregate existing aggregates, and (5) neutralize and/or remove existing aggregates. The first generation of drugs tested in clinical trials focused on reducing Aβ production by inhibiting the enzymes which produce Aβ, and by targeting Aβ aggregates using antibodies. While the latter approach has proved successful, the former has not.

All the anti-Aβ monoclonal antibodies (mAbs) either approved (e.g. lecanemab, donanemab, aducanumab) or currently being tested in clinical trials (e.g. trontinemab, remternetug, sabirnetug, PRX012, ALIA-1758) preferentially bind to aggregated forms of Aβ. Nonetheless, important differences exist between these antibodies. Lecanemab, aducanemab, sabirnetug, and trontinemab preferentially recognize aggregated forms of Aβ, and retain the ability to engage de novo aggregates after plaques^1^ are removed. Donanemab, remternetug, and ALIA-1758 are distinct because they preferentially recognize Aβ with a pyroglutamate 3 (pE3) N-terminus. pE3-Aβ is formed after Aβ deposits in plaques and is not anticipated to be present in early aggregates formed after plaques are removed. Thus, while antibodies which target pE3-Aβ are effective at removing pre-existing plaques [9, 27], they are not anticipated to engage with de novo formed soluble aggregates and plaques [28]. Careful analysis of patients that respond versus those who do not respond to Aβ immunotherapy is required to distinguish between antibody specific effects and more broad biological reasons underlying the poor response in certain individuals. The latter, include ApoE driven effects and the presence of co-pathologies which may not be altered by anti-Aβ treatments.

Besides optimizing target engagement, effective immunotherapies need to minimize the most prevalent adverse effect seen with anti-Aβ antibodies—amyloid-related imaging abnormality (ARIA) [29]. ARIA is categorized into two types: (i) vasogenic edema or effusion, (ARIA-E) and (ii) hemosiderin deposition (ARIA-H) [30]. Most cases of ARIA are asymptomatic, and in those with symptoms, the presentation often self-resolves even when drug administration is continued; however, a small percentage of cases are serious and a very small number are fatal [8, 9, 31].

Trontinemab differs from the current antibodies in clinical trials in that it appears to facilitate the very rapid clearance of plaques with little ARIA [32]. Trontinemab incorporates the same paratopes as gantenerumab – an antibody that failed to meet its primary endpoint in two Phase 3 trials, but slowly cleared amyloid and showed positive trends on several clinical outcomes [33]. Trontinemab is a bispecific fusion protein that differs from gantenerumab and other clinical anti-Aβ antibodies due to the incorporation of an anti-transferrin receptor (TfR) motif which facilitates blood–brain barrier (BBB) crossing and increases brain exposure [34, 35]. After only three monthly infusions of 1.8 mg/kg, 36% of patients had complete removal of amyloid as detected by PET imaging; after 7 infusions, 75% of patients were “amyloid negative” (< 24 centiloids) at week 28. Importantly, this rapid effect was achieved at a tenth of the monthly dose of lecanemab or donanemab. In addition, the incidence of ARIA was extremely low, with only 1 out of 16 patients (4 placebo and 12 drug) in the 1.8 mg/kg group, and no cases in the 3.6 mg/kg group (4 placebo and 8 drug). Interim analysis of a larger 1.8 mg/kg group (12 placebo and 48 drug) who received an average of ~ 5 doses had 2 cases of ARIA-E. In the 3.6 mg/kg group (35 combined placebo and drug) who received an average of ~ 3 infusions no cases of ARIA-E were observed but 5.7% of subjects had evidence of ARIA-H [32]. Recent mouse studies suggest that TfR-mediated transfer of anti-Aβ mAbs across the BBB mitigates ARIA-like lesions by facilitating broad brain parenchymal distribution while avoiding arterial perivascular spaces where CAA is common [36].

If the results of the trontinemab Phase 1b/2a are confirmed in the forthcoming Phase 3 trial, it could transform the future treatment paradigm for patients with AD by facilitating a short treatment period with immunotherapy (~ 3–6 months), which ideally would be followed by maintenance treatment, such as an oral small molecule or vaccine, to prevent re-accumulation. Moreover, if TfR shuttling mitigates risk for ARIA, this in turn could negate the need for regular magnetic resonance imaging (MRI) monitoring [31], and thus significantly reduce the burden to patients, providers, and payers.

Beyond BBB shuttles, the use of gene or cell therapy to deliver anti-Aβ antibodies hold great potential. A major advantage of this approach over Aβ vaccines is the prospect of expressing clinically validated antibodies with established efficacy and safety profiles. However, if the antibodies employed have risk for ARIA, it will be important to make expression controllable so that antibody production can be turned off. On the other hand, this may not be necessary if BBB shuttles are incorporated into the expressed antibody.

As has been established in preclinical studies, vectorized anti-Aβ antibodies could be delivered via a single intramuscular administration [37, 38], leading to long-lasting therapeutic levels of the antibody. Such an approach would revolutionize passive immunotherapy and offer considerable advantages beyond trontinemab and other next-generation antibody approaches. Not least, a “one-and-done” administration could be the holy grail for primary and secondary prevention. Cell therapy approaches that allow controllable production of an encoded mAb are an alternative means to achieve the same ends as vectorized mAbs. In this regard, allogeneic B cells, which can be engineered to produce an anti-Aβ antibody, are of particular interest. Such approaches would lessen patient burden and cost to payers and merit detailed investigation.

As seen in the recent COVID epidemic, vaccines have considerable advantages for primary prevention in large populations, and this should also be true for AD. Indeed, the first immunotherapy trials in AD used an active Aβ vaccine (AN-1792) [39–41], which in patients who developed anti-Aβ antibodies achieved clearance of plaques [42, 43]. Unfortunately, testing of AN-1792 was discontinued after ~ 6% of dosed patients in a Phase 2 trial developed meningoencephalitis [41]. Of the 300 patients who received drug approximately 20% developed antibodies, and responders who subsequently came to autopsy evinced long-lasting clearance of amyloid pathology [44]. Attenuating unwanted T-cell responses, maximizing the number of responders vaccinated and antibody titer, and tuning the response to preferentially recognize aggregated Aβ without inducing ARIA remain significant challenges for the promise of Aβ vaccination to become a reality. Currently, only a handful of active vaccine candidates are in development (Table 1), but clearly this is an area that deserves further effort.

**Table 1:**
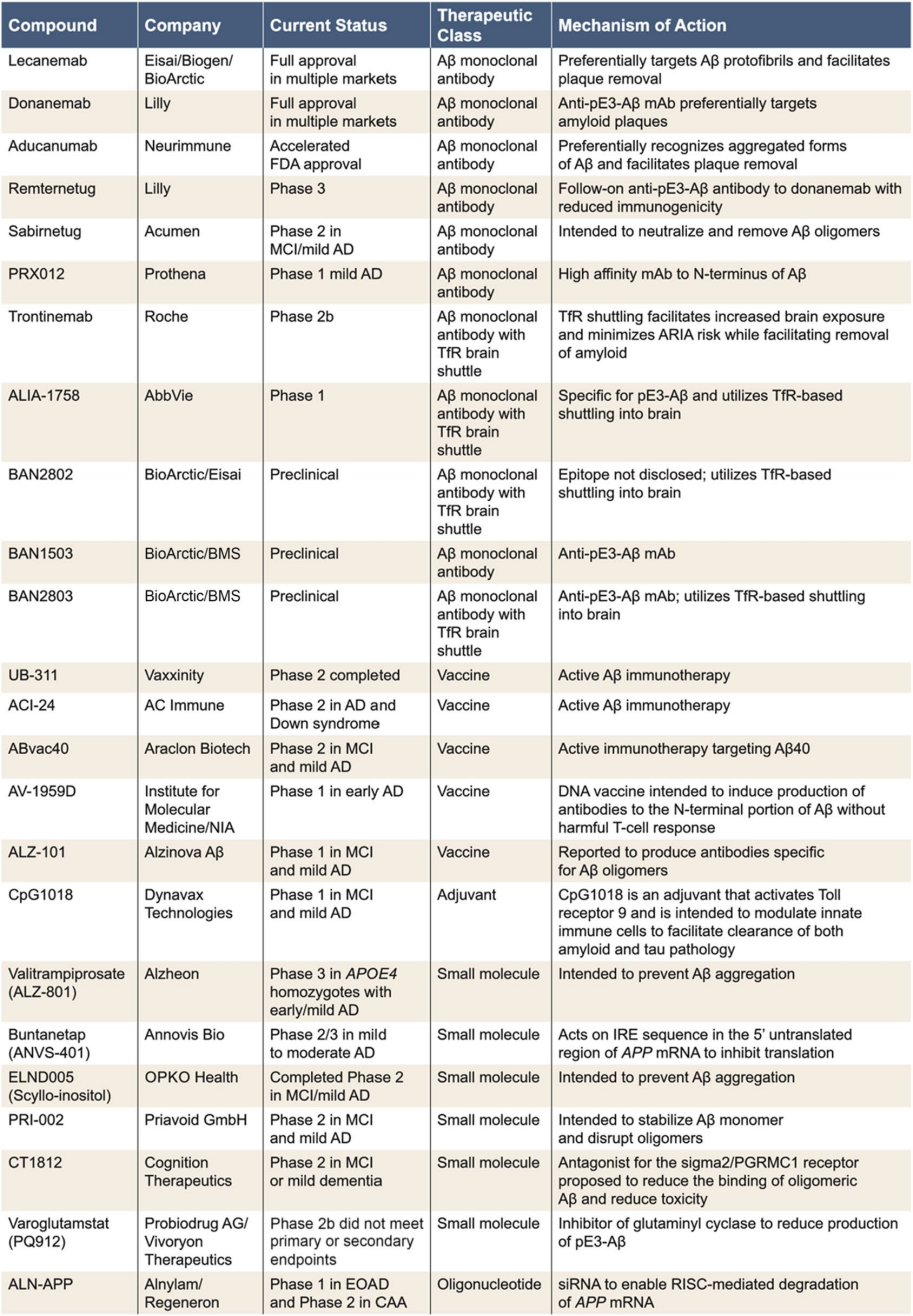
Current and recently terminated Aβ programs

The list of preclinical programs is not meant to be exchaustive, but rather to illustrate the extent of effort underway and the defferent modalities being deployed

*ARIA* amyloid-related imaging abnormalities, *CAA *cerebral amyloid angiopathy, *EOAD *early-onset Alzheimer's disease, *IRE *iron-response element, *mAb *monoclonal antibody, *MCI *mild cognitive impairment, *mRNA *messager RNA, *NIA *National Institute on Aging, *pE3 *pyroGlu3, *PGRMC1 *progesterone receptor membrane component 1, *RISC *RNA inducing silencing complex, *siRNA *small interfering RNA, *TfR *transferrin receptor

Primary prevention could also be facilitated by the development of effective, safe, oral therapies which target the earliest stages of Aβ dyshomeostasis [4, 45]. To date, the most explored approaches have targeted Aβ production. Five β-secretase inhibitors (BACEi) were terminated in late-stage clinical trials [46–49]. Most BACEi were tested in amyloid-positive patients with mild/moderate AD, two were tested in early/mild (Stages 2–3) AD [50, 51]. None showed clinical benefit, and doses which caused > 60% reduction in cerebrospinal fluid (CSF) Aβ were associated with transient cognitive worsening [48, 51–54]. Even when administered for 24 months, change in amyloid PET was minimal (< 6%). Thus, reduction of Aβ monomer levels appears to have limited impact on pre-existing Aβ aggregates. Similarly, results from a trial using an antibody for Aβ monomer (solanezumab) evinced no clinical benefit and only modestly attenuated the rate of amyloid accumulation. This despite the dose being predicted to engage ~ 50% of central nervous system (CNS) Aβ monomer. The fact that BACEi treatment which reduced Aβ levels by > 65% modestly reduced amyloid [54], whereas solanezumab attenuated the rate of accumulation but did not reduce amyloid, suggests that > 50% engagement of target is required to remove amyloid. Moreover, the BACEi data indicate that even greater reduction of monomers will not yield benefit in individuals with substantial pre-existing amyloid. However, targeting monomer production would be expected to have benefit if given to individuals before amyloid accumulates and deposits, or as a maintenance therapy after amyloid has been removed. The challenge for the BACEi approach is to demonstrate that doses capable of reducing Aβ by ~ 30% (the target predicted by the protective APP mutation A673T) are unambiguously safe. This would necessarily require long duration safety studies which may not be commercially feasible.

Inhibiting γ-secretase, the other protease required for Aβ production, was tested and abandoned due to on-target toxicity [55, 56]. Notably, γ-secretase is one of only a handful of intramembrane cleaving proteases and serves an essential function releasing more than 200 substrates from the membrane [57], and in the case of Notch, the intracellular fragment that is released serves a critical physiological role [58]. A key feature of γ-secretase is its processive cleavage of the APP-C-terminal fragment (known as CTFβ or APP-C99). Initial γ-secretase cleavage occurs at the ε-sites to generate Aβ49 and AICD50, or Aβ48 and AICD49. γ-secretase then sequentially trims the C-terminus of the N-terminal fragments in ~ 3 amino acid intervals to generate Aβ49->46->43->40->37 or Aβ48->45->42->38. Through the use of cryo-electron microscopy and functional studies on purified γ-secretase complexes, it is now understood that AD-causing mutations in presenilins destabilize γ-secretase–APP interactions, leading to premature release of longer, aggregation-prone Aβ peptides (e.g., Aβ43 and Aβ42) [59]. Conversely, certain small molecules which bind γ-secretase can stabilize the enzyme–APP complex, reducing production of longer forms of Aβ while increasing shorter alloforms, including Aβ37 and Aβ38 [60]. Unlike γ-secretase inhibitors which block the necessary cleavage of a burgeoning list of substrates, γ-secretase modulators (GSMs) effect the processivity of the enzyme, and therefore should not impede the physiological and potentially important signaling mediated by γ-secretase cleavage of other substrates. Small orally available molecules with suitable properties have the potential for use in primary prevention, early secondary prevention and as a maintenance therapy for amyloid-depleted AD patients who have already achieved plaque clearance with anti-Aβ immunotherapy. For early secondary prevention, patients should have amyloid levels lower than in the Solanezumab failed A4 trial, where the predicted reduction of total Aβ levels of ~ 50% did not remove amyloid. A potential downside of GSMs may result from elevation of Aβ38 and Aβ37, which are enriched in CAA [61].

Currently, RG6289 is the only GSM in active clinical trials. Data from a Phase 1, single/multiple ascending dose trial in healthy volunteers indicates that RG6289 is highly potent and selective, with a favorable safety and tolerability profile, and a pharmacokinetic (PK) profile supporting daily administration [62]. RG6289 is currently in Phase 2 recruiting amyloid positive unimpaired or Stage 3 AD subjects with a global CDR of 0 or 0.5.

Besides targeting the enzymes which generate Aβ, another approach to reducing Aβ production is to lower the expression of APP, the parent molecule from which Aβ is derived. This could be done at multiple levels, including by inhibition of transcription and translation. In this regard, the Alnylam ALN-APP small interfering RNA (siRNA) program is the most advanced. Recent interim results from a Phase 1 trial of 20 patients demonstrated dose-dependent suppression of CSF APPs and Aβ, which was sustained for 6 months after a single intrathecal administration of ALN-APP [63]. These encouraging results supported the initiation of a Phase 2 trial, which began in May 2024 in adults with sporadic CAA and Dutch-type CAA (NCT06393712). While intrathecal administration is not favored by patients, and could be a barrier for primary or secondary prevention, it may be possible to develop oligonucleotides with improved PK that support even less frequent intrathecal dosing, or to utilize BBB shuttle technology to enable intravenous delivery. These possibilities further emphasize the importance of technical innovation to attain optimal drugs for validated targets.

In addition to targeting Aβ production, attention should be given to enhancing Aβ degradation and/or to preventing or disrupting Aβ aggregates, preferably using small molecule approaches. Currently, there are no publicly disclosed commercial programs aimed at enhancing degradation of Aβ, and there are limited efforts at developing small molecules to prevent aggregation or destabilize already formed aggregates (Table 1).

Valiltramiprosate (ALZ-801) is a prodrug of homotaurine, which in various forms has been in development for almost 20 years, and is claimed to prevent the aggregation of Aβ42 into larger toxic aggregates (Table 1). ALZ-801 was tested in a Phase 3 trial in *APOe*4 homozygotes with mild AD or mild cognitive impairment (MCI) due to AD [64], and the results were presented at the recent ADPD conference. The trial was unique in that the inclusion criteria allowed enrollment of patients with radiographic evidence of microhemorrhages and superficial siderosis. Unfortunately, while the drug was safe, the trial failed to meet its primary and secondary endpoints.

Glutamyl cyclase is a metalloprotease capable of catalyzing the conversion of N-terminal glutamic acid (E) residues to pyroglutamate (pE) and can convert E3-Aβ to pE3-Aβ. This modified form of Aβ is a component of amyloid plaques in the human brain, and the primary target for donanemab, remternetug and earlier stage mAbs (Table 1) [65]. pE3-Aβ is more prone to aggregation and is thought to be more toxic than unmodified Aβ [66, 67]. In mouse studies reducing glutaminyl cyclase activity reduced amyloid deposits and attenuated cognitive impairment in APP transgenics [68, 69]. However, varoglutamstat, a small molecule inhibitor of glutamyl cyclase failed to meet its primary and secondary endpoints in a Phase 2 trial in participants with MCI and mild AD [70, 71]. It should be noted that preventing pE-Aβ formation is quite distinct from using antibodies that enable microglial-mediated removal of pE-Aβ-containing plaques. Specifically, inhibiting the conversion of E3-Aβ to pE3-Aβ is not expected to remove pre-existing plaques, but rather slow down accrual and possibly decrease the nucleation of new plaques.

Clearly, there is considerable scope to exploit evolving biological understanding and technological innovations to develop small molecules that enhance Aβ degradation and clearance or inhibit its production, but much remains to be done. This might also include revisiting previously terminated programs or repurposing FDA approved drugs.

### Patient population and biomarkers consideration for Aβ/APP targeting interventions

Current anti-Aβ therapies are approved for AD Stages 3 and 4, however, given the initiating role of Aβ in disease it is anticipated that early intervention will deliver maximal efficacy, hence the ultimate goal is to apply Aβ-targeting therapies for primary (Stages 0 and 1) and secondary (Stage 2) prevention. Developments in the biomarkers field have revolutionized the study of AD and are key to identifying early patient populations, monitoring disease progression and assessing therapeutic effects of investigational drugs. For most Aβ/APP targeting interventions there already exist suitable biomarkers for patient selection and target engagement (amyloid PET, CSFAβ42/40, CSF phospho-tau/tau, plasma phospho-tau and tau PET), and safety biomarkers are in place for immunotherapy approaches (MRI for ARIA and anti-drug antibody immunogenicity assays) [72]. Protype CSF assays are also available to measure specific Aβ alloforms (e.g. Aβ37, Aβ38 and pE3-Aβ) [73] and aggregation states of Aβ [74, 75] and to measure changes in secreted APP [63]. Excitingly, the FDA recently approved the first in vitro diagnostic blood-based device to aid in diagnosing AD—the Lumipulse G pTau217/β-Amyloid 1–42 plasma ratio [76], and application of plasma pTau217 screening in an ongoing Phase 2 trial was recently reported to dramatically reduce the requirement for amyloid PET [77]. This movement to blood-based diagnostics has great potential for facilitating faster and more const effective trials, and most importantly, for allowing identification of early stage disease in real world settings.

## Targeting tau

Unlike amyloid deposits that appear diffusely throughout the brain for up to 20 years before the onset of AD symptoms, the temporal and spatial appearance of tau deposits are more closely related to where brain atrophy occurs and cognitive deficits originate [15, 16]. Indeed, natural history studies using tau PET imaging demonstrate that in life the evolution of tau pathology correlates well with the emergence of clinical symptoms [18]. Interestingly, in trials of anti-Aβ antibodies which slowed cognitive decline, the levels of certain forms of phospho-tau were decreased and, in most cases, so were the relative rates of tau PET signal [8, 19, 78]. Moreover, in rare cases of resilience to autosomal-dominant AD, individuals had profound amyloid accumulation, but minimal and late-evolving tau pathology [79, 80]. Collectively, these human data demonstrate the importance of tau accumulation in driving symptomatic presentation. However, tau is not a simple target.

**Fig. 3:**
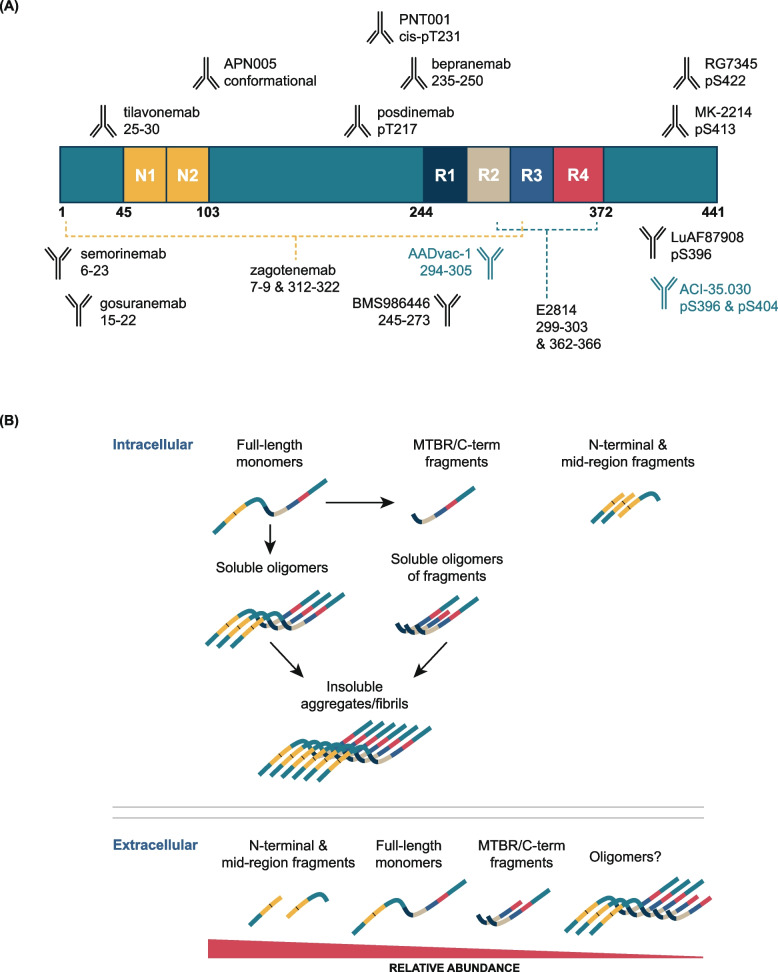
Domain structure of tau, epitopes of antibodies used in recent and current clinical trials, and schematic depicting the molecular heterogeneity which characterizes the pools of tau inside and outside of neurons. **A** There are six major isoforms of tau in human brain. Differential splicing of the R2 domain within the microtubule binding region (MTBR) gives rise to three 3R isoforms (2N3R, 1N3R, 0N3R) and three 4R isoforms (2N4R, 1N4R, 0N4R). Two hexapeptide motifs ^275^VQINK^280^ in R2, and ^306^VQIVYK^311^ in R3, are essential for aggregation, thus aggregation-competent fragments of tau are defined by the presence of one of these. The first anti-tau immunotherapies tested in the clinic recognized N-terminal epitopes (semorinemab, gosuranemab, tilavonemab, zagotenemab) and have been discontinued. Current anti-tau monoclonal antibodies and vaccines are targeting epitopes in and around the MTBR domain. **B** Domains of full-length tau and tau fragments are colored as in (**A**). Inside healthy neurons tau exists primarily as unaggregated full-length proteins whose interactions with microtubules are regulated by phosphorylation. Small amounts of tau fragments are also present, but the processes by which these are generated and whether they have a function is not understood. Outside neurons the most abundant forms of tau are N-terminal and mid-region fragments, a portion of which are phosphorylated, and some of which is contained in microvesicles. Minute amounts of aggregation-competent tau are also present in free solution and within microvesicles. During disease, full-length tau and aggregation-competent fragments of tau oligomerize and subsequently form insoluble aggregates which display a myriad of post-translational modifications including: truncation, hyperphosphorylation and ubiquitination. As disease progresses aggregates of tau become abundant within certain neurons. In CSF the levels of extracellular N-terminal and mid-region tau fragments increase initially and then plateau, whereas the levels of aggregation-competent forms of tau (although very low) continue to increase during the course of disease

Tau is encoded by the *MAPT* gene, and in human brain, there are six different splice isoforms which give rise to proteins of 352–441 amino acids (Fig. 3A). Each isoform can undergo a variety of post-translational modifications, including phosphorylation (of which there are ~ 80 unique sites), ubiquitination, sumoylation, O-GlcNAcylation, acetylation, glycosylation, isomerization and proteolytic cleavage [81, 82]. Inside neurons, tau largely exists as full-length monomers and C-terminal fragments, whereas outside neurons, N-terminal and mid-region fragments predominate [83, 84] (Fig. 3B). Thus, there are a considerable number of full length tau monomers or microtubule binding region (MTBR)-containing fragments which are competent to aggregate. These in turn can form a range of species from soluble “readily diffusible” oligomers to large insoluble end-stage aggregates (Fig. 3B). Moreover, there is mounting preclinical evidence that extracellular MTBR-containing fragments of tau are directly synaptotoxic [85, 86]. In addition to gains of toxic activity, there is compelling preclinical evidence that tau can act as an enabler of toxicity, such that knock-down or genetic ablation of tau protects against a variety of neurotoxins [87–90]. In our view, it is likely that tau is both a mediator and a facilitator of toxicity, with the mediation of toxicity dependent on aggregation. Thus, aside from the greater molecular complexity of tau compared with Aβ, the potential strategies for drugging tau are highly similar to those for Aβ (Fig. 2).

Currently, there are programs targeting each of the five intervention points illustrated in Fig. 2 (Table 2). The majority of recent trials have tested or are testing antibodies that recognize a variety of different epitopes across the tau primary sequence (Fig. 3A). These immunotherapy approaches are based on the so-called “spread hypothesis”, which posits that aggregation-competent forms of tau can pass from donor neurons to seed and “spread” aggregation in recipient neurons [91]. Since tau lacks the signal sequences necessary for conventional secretion, “spread” could only occur by non-conventional or non-specific mechanisms [92]. Non-conventional mechanisms include release in exosomes and transfer through thin membranous bridges termed tunnelling nanotubes. If transfer were to occur by such mechanisms, then tau may not be accessible to antibodies. However, since tau is one of the most abundant proteins in brain, it is conceivable that seeds could leak into the extracellular space during neurotransmission or secondary to synaptic/cellular compromise. In this case, seeds could potentially be engaged by antibodies. However, physical spread of tau aggregation remains an unproven hypothesis, and even if correct, the molecular identity of the “seeding” entities and their concentrations are unknown.

**Table 2:**
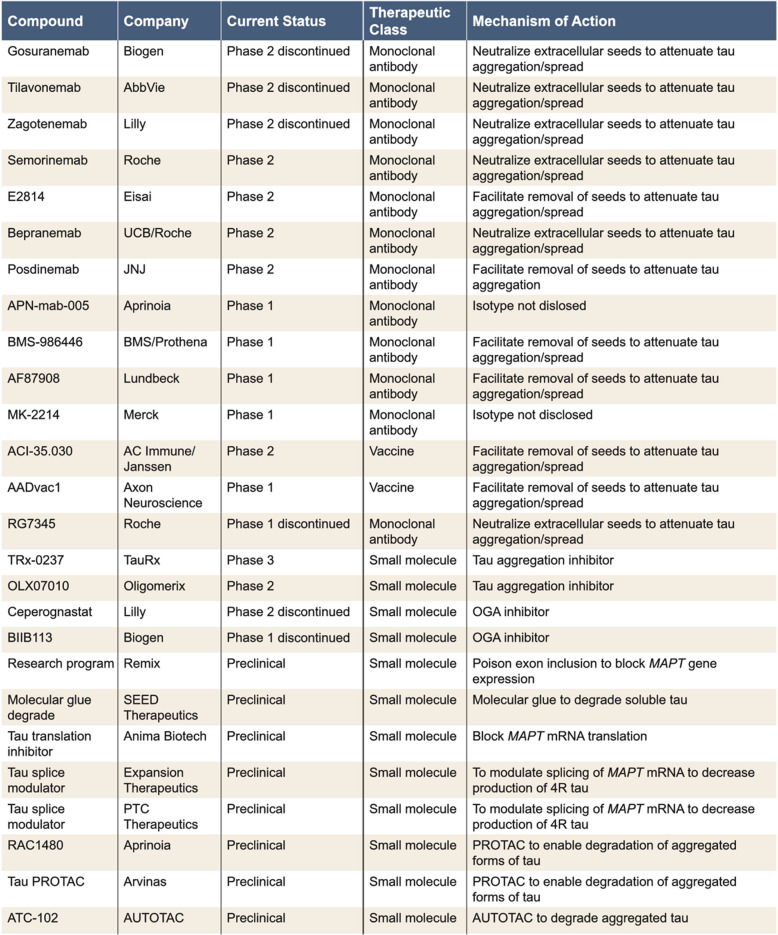

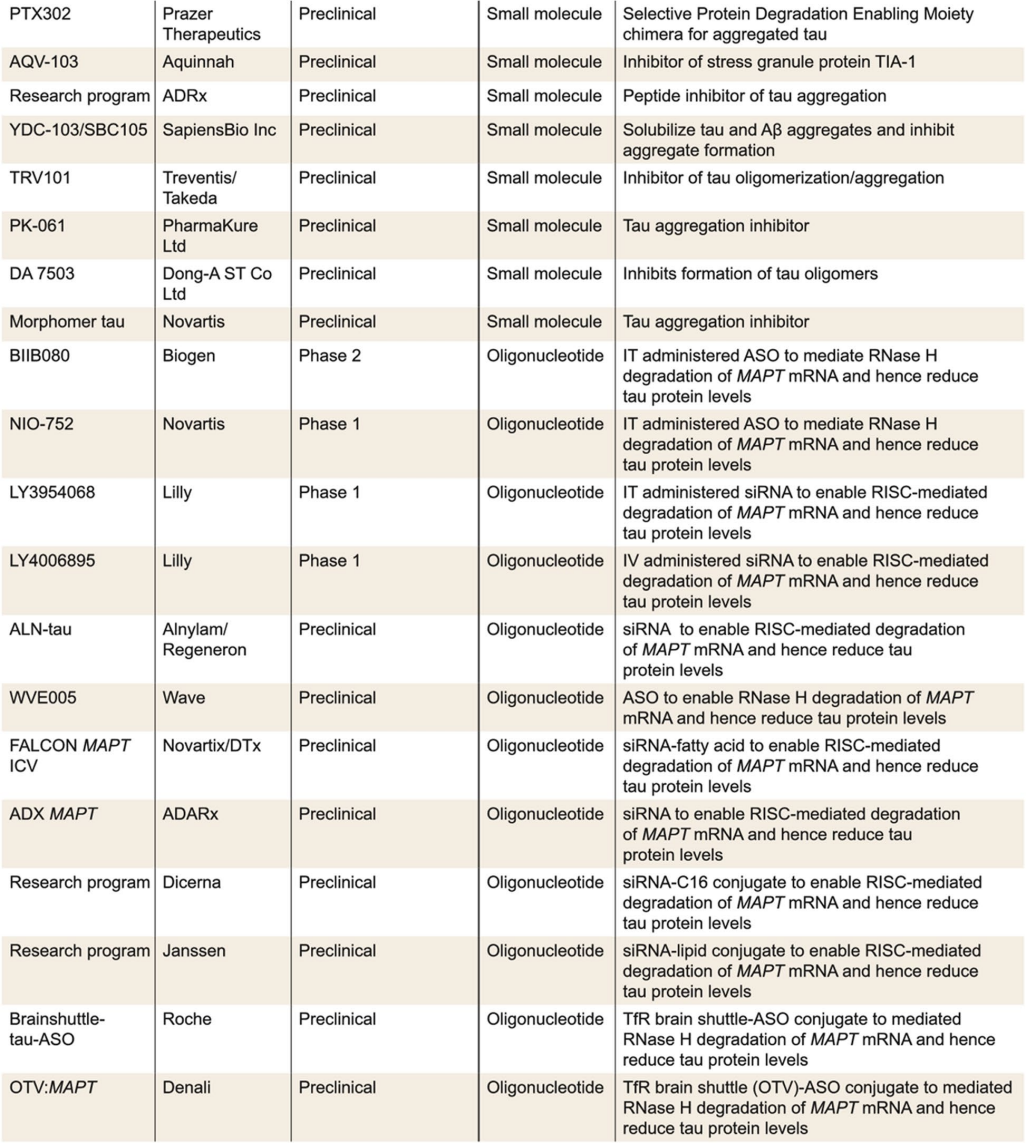
Current and recently terminated tau targeting programs

The list of preclinical programs is not meant to be exchaustive, but rather to illustrate the extent of effort underway and the defferent modalities being deployed

*ASO *antisense oligonucleotide, *AUTOTAC *autophagy-targeting chimera, *IT *intrathecal, *IV *intravenous, *MAPT *misrotubule-associated protein tau gene, *mRNA *messenger RNA, *OGA *O-GlcNAcase, *OTV *oligonucleotide transport vehicle, *PROTAC *proteolysis-targeting chimera, *RISC *RNA inducing silencing complex, *siRNA *small interfering RNA, *TfR *transferrin receptor, *TIA *T cell intracellular antigen-1

**Fig. 4:**
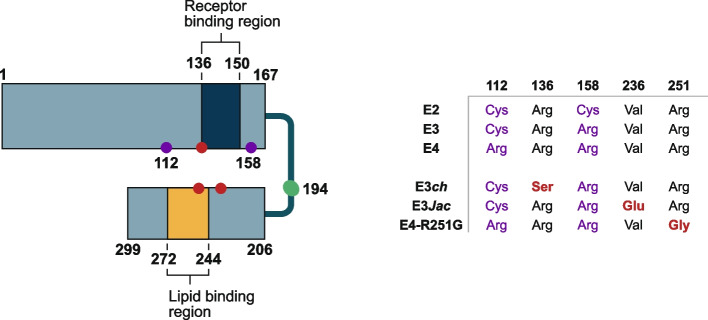
Domain structure of ApoE and amino acid differences for common and rare variants. ApoE has three major domains, its N-terminal (1–167), hinge region (168–205), and C-terminal (206–299). The receptor binding region (136–150) and lipid binding region (244–274) are shown in dark blue and yellow, respectively. Threonine 194, the site at which ApoE is glycosylated is denoted with a filled green circle. The position of residues 112 and 158 which differentiate ApoE2, ApoE3, and ApoE4 are indicated with filled purple circles, and rare variants at residues 136, 236 and 251 are denoted with red filled circles. The table on the left summarizes the amino acid differences between the common (purple font) and rare variants (red font)

To date, four anti-tau antibodies (gosuranemab, tilavonemab, zagotenemab, and semorinemab) have failed to demonstrate a clear clinical benefit in patients with early AD, nor have they demonstrated any impact on tau pathology [93–97]. Notably, all four failed antibodies have epitopes in the N-terminus of tau and therefore can only engage with aggregation-competent forms of tau which span from the N-terminus to the MTBR—such species are not abundant in human fluids [98] (Fig. 4). However, certain fragments of tau which contain all or part of the MTBR have been identified in human fluids and brain tissue [99, 100]. If aggregation of tau does proceed via transfer of aggregation-competent species in the extracellular milieu, antibodies which target in or near the MTBR should attenuate progression of tau pathology. Unfortunately, the results from a recent Phase 2 study of bepranemab, an antibody which recognizes an epitope just N-terminal to the MTBR, have been disappointing [101]. Bepranemab failed to reach its primary clinical endpoint, and its effects on tau PET, although suggestive of a treatment effect, are confounded because more patients in the placebo group had high tau levels than in the treatment groups [101]. Moreover, its effects on secondary and exploratory endpoints were mixed, and those that were positive were of relatively low magnitude.

Two other MTBR targeting antibodies are currently being tested in humans: E2814 and BMS-986446. E2814 recognizes the same heptapeptide epitope present in R2 and R4 of the MTBR domain (Fig. 3A). It is well tolerated (NCT04231513) and has been shown to engage with a soluble biomarker of tau aggregates (MTBR243) [102], but its effects on tau PET have been reported for only 3 participants, and the results were mixed [103]. Thus, at the time of writing, the jury is still out on whether targeting the MTBR region of tau with antibodies will have benefit, or if the potential benefit will be greater than that attained with anti-Aβ immunotherapy.

Due to the limited understanding of the locations and forms of tau that should be targeted, we recommend an approach that will reduce all forms of tau in all locations. This is feasible because substantial reduction in tau expression in both mice and humans is without adverse effect. In mice, complete knockout of tau is well tolerated, with only mild phenotypes emerging with old age in some [104–106], but not all, knockout models. Moreover, prolonged suppression of tau in adult mice is without adverse effect [107]. Most importantly, analysis of the UK BioBank identified 230 individuals with predicted loss-of-function (LOF) mutations in *MAPT*, and these were without any apparent clinical consequence (Chris Whelan Personal Communication).

In principle, tau lowering could be achieved by directly targeting tau at the genome, transcriptional or protein level, or indirectly by modulating targets which modify tau expression. The concept of tau lowering has been greatly boosted by results from the recent Phase 1b study of the *MAPT* antisense oligonucleotide (ASO), BIIB080. Exploratory analysis provided the first-in-human demonstration that suppression of tau expression can reduce PET-detectable tau aggregates across multiple brain regions [108]. Moreover, this occurred after as little as 6 months, and prolonged suppression of tau was well tolerated [109]. Intriguingly, other exploratory analyses showed numerical differences favoring high-dose BIIB080 on multiple cognitive and functional scales in both the multiple ascending dose and long-term extension phases of the trial [110]. Together, these preliminary data provide the first evidence that lowering tau expression appears to have an acceptable safety profile, can decrease tau pathology, and may have clinical benefit. However, the sample size in the BIIB080 study was small, and external controls were used for the long-term extension arm. Confirmation of clinical benefit will require larger studies, such as the ongoing Phase 2 CELIA study (NCT05399888) and studies by other sponsors also employing *MAPT* RNA-lowering oligonucleotides (NCT04539041, NCT06297590, LY3954068, Table 2). Similarly, it will be important to determine if the increase in ventricular volume [108, 109] and in CSF NfL levels [109] in BIIB080 Phase 1b treatment groups are replicated in larger studies, and if they are, whether they have any clinical consequences.

A potential drawback of using standard oligonucleotides is the need for intrathecal administration, which may limit patient enthusiasm. Thus, targeting *MAPT* RNA to achieve tau lowering with modalities that afford greater patient convenience should be a priority for the field. Fortunately, there are several ways to enable peripheral administration of oligonucleotides. These include the use of oligonucleotide-brain shuttle conjugates [111, 112] and lipid nanoparticle (LNP) [113], or exosomes [114] which can be “addressed” to deliver oligonucleotides to the brain. The use of lipid nanoparticles conjugated with anti-TfR antibodies to deliver oligonucleotides to the brain is particularly appealing since it would allow systemic delivery of a clinically proven molecule. However, the use of lipid nanoparticles for brain delivery is still in its infancy [113].

Suppressing tau transcription via zinc-finger proteins (ZFP), TALEN, CRISPR, or related technologies have the potential to achieve robust tau reduction with a high degree of precision. Such approaches are attractive since they could be “one and done” treatments, but considerable technical advances are required to enable the necessary broad CNS distribution.

Mechanistically, degradation of tau monomer should produce results comparable to suppression of tau expression, but with the potential advantage of an orally available drug. This could be accomplished using small molecules that direct tau to a cell-intrinsic degradation pathway (e.g. proteasome, autophagosome). Molecular glues are small molecules that function by converting a target protein into a “neo-substrate” for an E3 ligase [115, 116] and subsequent targeting to the proteasome for degradation. While this approach has been successfully implemented for non-CNS targets [115, 117], its feasibility for CNS targets (and particularly for tau) requires exploration (Table 2).

Besides decreasing tau monomer levels, the next most appealing approach is to target the removal or disruption of pre-existing tau aggregates. This strategy will not address the potential “toxic facilitator” role that tau monomer appears to play, but it should ameliorate the toxicity of tau aggregates and their potential for proliferation between cells. Conceivably degradation of tau aggregates could be achieved using heterobifunctional small molecules, with a warhead to specifically bind aggregates and a moiety to direct the complex to a cell-intrinsic degradation pathway (e.g. proteasome, autophagosome) [118, 119]. Proteolysis-targeting chimeras (PROTACs) shuttle target proteins to the proteasome for degradation, whereas autophagy-targeting chimeras (AUTOTACs) facilitate degradation by delivering target proteins to autophagosomes, and there are several early-stage programs exploring the use of these technologies (Table 2). A concern about the use of PROTACs, molecular glues, and AUTOTACs, is that the proteasome/autophagosome systems may already be dysfunctional and shuttling more substrates to them may not be productive and could even be deleterious.

As with Aβ, inhibiting tau aggregation and/or facilitating the disassembly of tau aggregates has been explored for a considerable time. Current programs use small molecule inhibitors which interact directly with tau aggregates (TRx-0237, OLX07010) and those which target enzymes whose activity impacts tau’s ability to aggregate (O-GlcNAcase [OGA] inhibitors). O-linked N-acetylglucosamine (O-GlcNAc) is a common post-translational modification of tau which is decreased in AD and in mice has been shown to reduce tau aggregation [120, 121]. O-GlcNAcase (OGA) removes O-GlcNAc from proteins and inhibition of OGA has been shown to attenuate tau pathology in several tau transgenic mouse lines, both in prevention and treatment paradigms [122–125]. Unfortunately, Lilly recently terminated the development of their OGA inhibitor ceperognastat after it failed to show benefit on a range of clinical endpoints [126]. The lack of benefit occurred despite the fact that ceperognastat produced favorable changes on key biomarkers, including attenuating brain volume loss, reducing the accumulation of PET-detected tau, and decreasing plasma p217tau [126]. Ceperognastat treatment was associated with dose-dependent serious adverse effects, which led the sponsor to stop development. While another OGA inhibitor remains in development (Table 2), whether the adverse effects seen with ceperognastat are molecule specific or generalizable to all inhibitors of OGA remains to be determined. OGA is known to act on > 100 different protein substrates [127, 128]; hence, and it may prove tricky to inhibit without unwanted side effects.

### Patient population and biomarkers consideration for tau targeting interventions

The temporal appearance and spatial distribution of tau deposits are closely related to where brain atrophy occurs and cognitive deficits originate [18]. Thus, disease-modifying, tau targeting therapies are expected to be most beneficial in early symptomatic patients when a high degree of function can still be preserved – primarily AD Stages 3 (MCI) and 4 (AD mild). However, it is anticipated that patients at even later stages of AD may also see cognitive benefit. Biomarkers should assist the identification of patient populations expected to benefit most from tau targeting therapies [129].

Various tau PET tracers are increasingly used in clinical trials evaluating disease-modifying therapies [8, 9, 108]. However, the tracers differ in affinity to aggregated tau, have different regional off-target retention, or have not yet been fully characterized. Efforts across academia and industry are underway to develop a universal scale for tau PET quantification of these tracers [130, 131]. Given the high cost and limited scalability of tau PET imaging, there is significant ongoing work to identify and progress fluid measures of brain tau pathology. Advancements in analytical methods have enabled the detection of multiple tau epitopes [129]. In particular, evolving data suggests that CSF MTBR-tau243 relates well to tau pathology [99, 132] and there is great interest in the endogenously cleaved, microtubule-binding region containing residue 243 (eMTBR-tau243) which can be measured in blood [133].

While it will be some time before approved blood-based markers of brain tau pathology emerge, fluid measures of soluble tau in the CSF (of which there are many different molecular forms, Fig. 3) have and are being used in clinical trials. For instance, soluble tau in CSF and blood have been used to assess target engagement following administration of anti-tau monoclonal antibodies and oligonucleotides (Table 2, e.g., [93, 94, 109, 134]). As discussed above, Tau PET has practical limitations, yet it is the most explored readout to assess pharmacodynamic response (e.g., [93, 94, 102, 109, 134]). In addition, there remains interest in FDG-PET and various markers of synaptic compromise (e.g., neurogranin and VAMP2) as distal indicators of pharmacodynamic effect.

Finally, besides amyloid driving tau mediated effects, there is also burgeoning evidence that the application of biomarkers of α-synuclein pathology will be critical to determine responders vs. non-responders of tau targeting therapies (see section below on ⍺-synuclein).

## Targeting ⍺-synuclein

Due to extensive overlap in the points of potential therapeutic intervention for tau and ⍺Syn (Fig. 2), and to avoid unnecessary repetition, this section will focus on the scientific premise for targeting ⍺Syn in AD, and briefly review current efforts and new approaches.

While ⍺Syn has long been linked with Parkinson’s disease (PD), AD was the first disease with which ⍺Syn was associated. Nine years after the isolation and identification of Aβ from amyloid plaques, two abundant non-Aβ peptide fragments were identified in purified plaques [135]. Antibodies raised to these peptides stained neuritic plaques, diffuse amyloid, and CAA. Subsequent cloning identified a 420 bp open reading frame encoding a 140 amino acid protein, which the authors dubbed “non-amyloid β-protein component of Alzheimer's disease amyloid” [135]. That protein is now better known as ⍺-synuclein. Succeeding discoveries have shown that ⍺Syn is the principal component of Lewy bodies (LB) found in a group of disorders referred to as synucleinopathies, which includes PD and dementia with Lewy bodies (DLB), as well as AD. Because of the impressive genetics linking ⍺Syn to PD and DLB, less attention has been paid to the role of ⍺Syn in AD. Nevertheless, compelling preclinical and clinical evidence implicate ⍺Syn and Lewy pathology in AD [136].

Several independent studies have found LB pathology to be the most common"non-classical AD"pathology detected in the brains of patients with late-onset AD, with estimates of prominent cortical Lewy pathology ranging from 33-42% of participants [137–139]. Deciphering whether cortical LB pathology is a part of the AD process, or a consequence of old age when many proteinopathies increase, is not trivial. However, analysis of brains from patients with AD causing mutations who died at relatively young ages is highly instructive. Notably, in certain studies, up to 50% of patients with familial AD had significant cortical LB pathology, whereas no other"non-classical AD"pathology was detected. Similarly, LB pathology is present in most patients with Down syndrome [140, 141]. At a minimum, these data suggests that ⍺Syn may play a role in a significant segment of patients with AD.

*In vivo* imaging of LB pathology is not yet possible and is an impediment to the development of ⍺Syn-targeting therapies. However, aggregates of ⍺Syn can be detected in human CSF using the synuclein amplification assay (SAA). Applying this assay to the analysis of specimens from the Biofinder natural history study revealed that 17–24% of patients with biomarker-confirmed AD Stages 3 and 4 were SAA-positive [142]. Similarly, assessment of AD clinical trial cohorts revealed that 25% of EMERGE/ENGAGE and 28% of TANGO patients were SAA-positive [143]. Moreover, elevated levels of total ⍺Syn in CSF have been detected in several AD cohorts, including a pre-symptomatic cohort of patients with familial forms of AD [144, 145]. Collectively, these observations imply that ⍺Syn may be involved early in disease pathogenesis, at least in a significant subset of patients.

SAA assessment in biomarker-confirmed (Aβ and tau) AD cases identified AD patient subgroups with specific symptoms and clinical progression profiles. Two recent independent studies showed that SAA+ AD patients had higher levels of visuospatial defects, hallucinations, and more rapid cognitive decline compared to SAA- AD patients [142, 146]. These data indicate that not only is it necessary to develop ⍺Syn-targeting agents to treat AD, but it will be important to determine how SAA+ patients respond to Aβ- and tau-targeting agents.

Similar to tau, ⍺Syn is one of the most abundant proteins in brain, can undergo multiple post-translational modifications, and exists in a range of different aggregation states [147]. Also like tau, ⍺Syn is produced inside cells, and although it lacks a conventional secretion signal, it is found in interstitial fluid, CSF, and blood. In contrast to tau, ⍺Syn further exists in both membrane-bound and unbound versions. Thus, the precise form or forms of ⍺Syn that contribute to disease remain uncertain; but, as with Aβ and tau, aggregation seems to be central to the pathogenic effects of ⍺Syn [148]. Accordingly, clinical and preclinical programs addressing the five intervention points described in Fig. 2 are being pursued. While these programs are primarily intended to develop drugs for PD or multiple system atrophy (MSA), they could be equally applicable to SAA+ AD patients, and we believe additional AD-specific effort is warranted.

The most advanced therapeutic programs targeting ⍺Syn employ antibodies designed to engage and remove aggregation-competent extracellular ⍺Syn (Table 3). The scientific premise for this approach is rooted in the hypothesis that aggregation-competent forms of ⍺Syn can pass from donor cells to recipient cells, and in so doing, propagate aggregation and pathology. However, since the precise nature of the propagating entity remains enigmatic, and its accessibility in the extracellular space is uncertain, targeting ⍺Syn with antibodies carries risk.

**Table 3:**
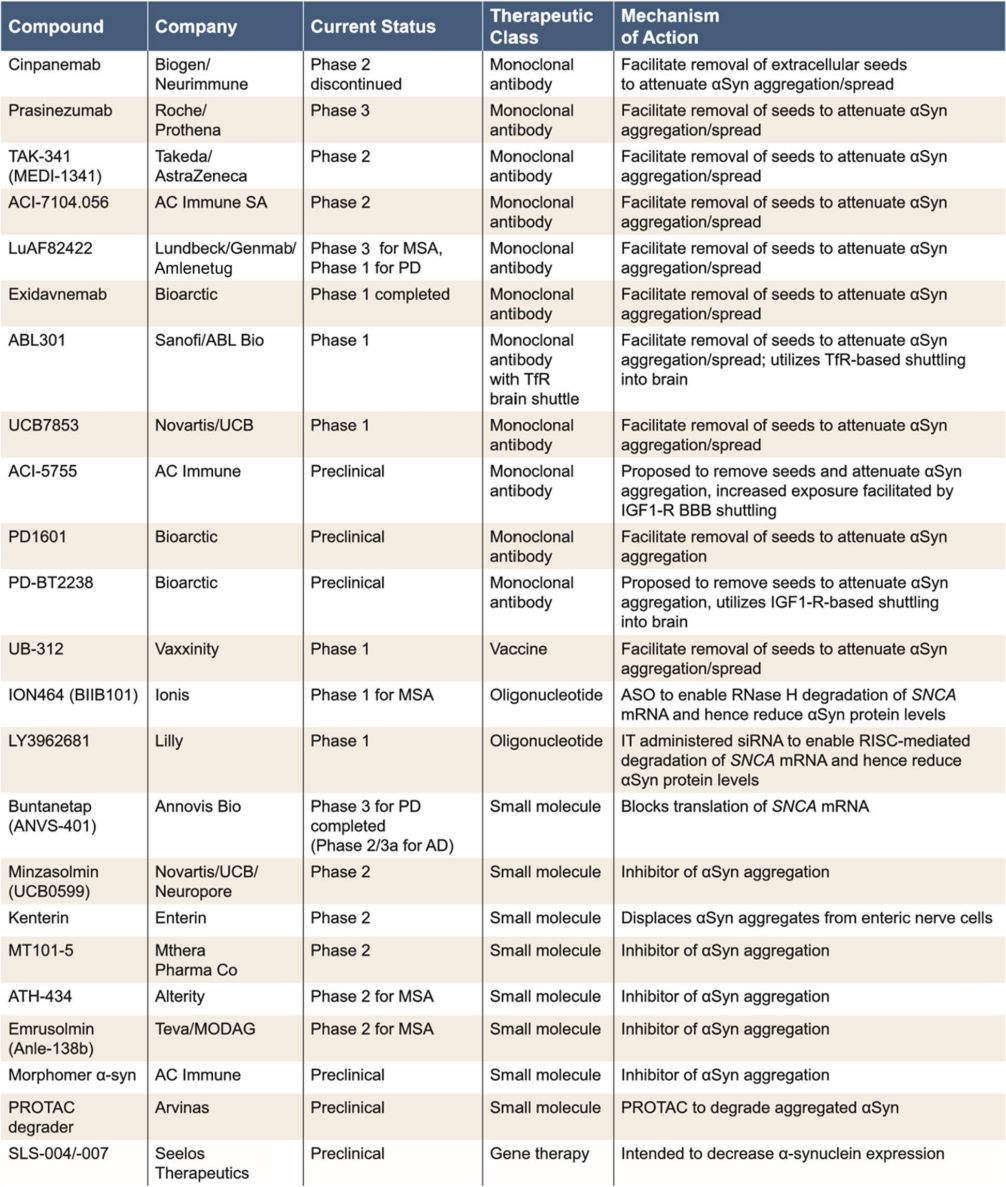
Current and recently terminated α-synuclein targeting programs

Programs are intended for PD unless otherwise indicated. The list of preclinical programs is not meant to be exchaustive, but rather to illustrate the extent of effort underway and the different modalities being deployed

*ASO *antisense oligonucleotide, *BBB *blood-brain barrier, *IGF1-R *insulin-like growth factor 1 receptor, *IT *intrathecal, *MSA *multiple system atrophy, *mRNA *messenger RNA, *PD *Parkinson's disease, *PROTAC *proteolysis-targeting chimera, *RISC* RNA inducing silencing complex, *siRNA *small interfering RNA, *SNCA* $\upalpha$ Syn gene, *TfR *transferrin receptor

Hints of efficacy have been reported in Phase 2 clinical trials of prasinezumab in PD [149–151] and Lu AF82422 in MSA [152]. Prasinezumab and Lu AF82422 target C-terminal epitopes in ⍺Syn and have considerably higher affinities for monomeric and oligomeric forms of ⍺Syn compared with cinpanemab, an N-terminal aggregate-preferring antibody which failed in Phase 2 [153–157]. The encouraging preliminary results with prasinezumab and Lu AF82422 suggest that targeting the C-terminus of ⍺Syn, which is proximal to the aggregation-prone non-amyloid-β component domain, may attenuate spread of pathology. Unfortunately, without the ability to reliably detect ⍺Syn aggregates in-life it is not yet possible to confirm the mechanism of action of these C-terminal targeting antibodies, and Phase 3 trials will rely solely on clinical endpoints. Clearly, development of an ⍺Syn PET ligand is a top priority for the field and is required to accelerate other ⍺Syn-targeting programs. Whether innovations such as the addition of BBB shuttles to anti-⍺Syn antibodies are required to achieve enhanced target engagement may be determined by biomarker analysis of the Phase 2 studies [158], but even if target engagement (measured in CSF) is deemed optimal for standard mAbs, it is reasonable to assume that TfR-mediated shuttling would ensure more widespread distribution of antibody in the parenchyma.

Despite the encouraging results with prasinezumab and LuAF82422, it is too early to declare success of ⍺Syn immunotherapy, and efforts with other modalities and mechanisms should continue apace. As with tau, we recommend approaches that do not require knowledge about the pathogenic forms of ⍺Syn or their site(s) of action. Here, we highlight modalities to lower total ⍺Syn levels, in particular and if possible, with orally available small molecule approaches.

Knockdown of ⍺Syn is plausible since studies of knockout mouse models demonstrate that peripheral ⍺Syn lowering is well tolerated [159], and in humans there appears to be redundancy between ⍺Syn and the other members of the synuclein family (β and γ). Moreover, it is known that ASO-mediated knockdown of ⍺Syn prevents the formation of new aggregates and partially clears existing ⍺Syn aggregates in mice [160]. These studies are reminiscent of preclinical tau-lowering studies [161] and give confidence that ⍺Syn lowering will be effective at removing pre-existing pathology in humans.

⍺Syn lowering could be achieved at the genome, transcriptional, or protein level using the approaches described in the previous section for tau. Here, we will briefly reprise information about the two approaches which are most likely to bear fruit in the nearer term, namely oligonucleotides, which suppress translation, and small molecule degraders (Table 3). Currently, ION464/BIIB101, an ASO, and LY3962681, an siRNA, targeting *SNCA* mRNA, are in Phase 1 trials (NCT04165486 and NCT06565195, respectively), and Alynlam have a siRNA program in early development [162]. Ideally, ASOs or siRNAs would be conjugated to BBB shuttles and optimized for their PK properties to facilitate infrequent intravenous delivery. With regard to degraders, we favor molecular glues over PROTACs since the ⍺Syn-binding warhead necessary for a PROTAC is difficult to design. This is due to the fact that ⍺Syn monomer is intrinsically disordered, and the structure of the putative tetramer is unknown. Conversely, while small molecules that bind ⍺Syn aggregates are available, it seems unlikely that the proteasome could degrade such macromolecular structures. In contrast, molecular glues offer a more promising approach because they have been shown capable of engaging with intrinsically disordered proteins such as MYC [163].

Currently, there are several molecules in clinical trials for PD or MSA aimed at inhibiting ⍺Syn aggregation. These molecules work through a variety of purported mechanisms, including inhibiting oligomeric ⍺Syn formation or by displacing ⍺Syn from the membrane (Table 3). In particular, molecules that inhibit oligomeric species are an attractive avenue for therapeutic development as increasing evidence demonstrates their toxicity in synucleinopathies [164]. However, limited data exist regarding the nature of oligomers in preclinical models compared with those in humans.

### Patient population and biomarkers consideration for ⍺Syn targeting interventions

Given that ⍺Syn pathology appears to contribute to disease progression in a sizeable proportion of AD patients there is concern that monotherapies targeting tau or Aβ in such patients will be of less benefit than in patients free of ⍺Syn pathology. Thus, an important near-term objective should be to determine whether patients with ⍺Syn pathology respond less well to approved anti-amyloid therapies, and to tau targeting therapies in development.

The use of the SAA as a highly sensitive tool (~ 95%) for confirming ⍺Syn pathology in the CSF of living patients [165] is advancing and prospective inclusion and exclusion based on ⍺Syn pathology (i.e., enrolling SAA + vs. SAA- patients) is expected to be available in the US in the near future. However, the current SAA assay has limitations. Most importantly, it is not suitable as a pharmacodynamic or prognostic indicator since the assay is binary (+ or -) and does not provide a quantitative determination of seed concentration. While there is considerable effort ongoing to develop quantitative SAA assays and to move to less-invasive matrices (e.g., skin and blood), it will be years before blood-based pharmacodynamic and prognostic markers of ⍺Syn pathology are approved. Similarly, while recent progress has been made in developing a PET ligand capable of detecting ⍺Syn aggregates in brain, it will be years before such ⍺Syn PET can be incorporated into clinical trials.

Separately, there are a number of useful markers for glia cells (GFAP and YLK) that have proven responsive to successful Aβ immunotherapy (e.g., [8]), and these could also be deployed in ⍺Syn targeting trials as distal pharmacodynamic markers. On the other hand, measurement of target engagement in CSF is well established [166] making it possible to advance early-stage trials of ⍺Syn lowering agents. Thus, while progress on pharmacodynamic markers is necessary, the current SAA assay should be sufficient for patient stratification, while direct measurement of ⍺Syn in fluids will allow assessment of target engagement by ⍺Syn lowering agents.

## Targeting ApoE

The role of *APOE* genotype in AD and its effect on amyloid have been recognized for more than three decades [167–169], but it is only relatively recently that ApoE’s involvement in other important AD pathways (including tau, ⍺Syn, vasculature integrity, and neuroinflammation) has been better recognized [79, 170–178]. As yet, there are a limited number of publicly disclosed drug development programs for *APOE*/ApoE (Table 4), consequently, we focus on the rationale for why and how to target *APOE*/ApoE with a brief commentary on current programs and discussion of future directions.

**Table 4:** Current *APOE*/ApoE targeting programs

Compound	Company	Current Status	Therapeutic Class	Mechanism of Action
NC181	NextCure	Pre-clinical	Monoclonal antibody	Removal of aggregated poorly lipidated ApoE
AEL-YO4	ADEL Pharma	Pre-clinical	Monoclonal antibody	Removal of aggregated ApoE4
7C11	Epoch Biotech	Pre-clinical	Monoclonal antibody	Inhibits ApoE binding to HSPG and potentially other receptors
AD-1801	BioArctic	Pre-clinical	Monoclonal antibody	Antibody targeting ApoE4; mechanism unclear
APOE ASO	IONIS	Pre-clinical	Oligonucleotide	ASO to enable RNase H degradation of *APOE* mRNA and hence reduce ApoE protein levels
APOE siRNA	Atalanta	Pre-clinical	Oligonucleotide	siRNA to enable RISC-mediated degradation of *APOE* mRNA and hence reduce ApoE protein levels
Obicetrapib	NewAmsterdam Pharma	Phase 3 (in HeFH)	Small molecule	CETP inhibitor to ameliorate ApoE4’s deleterious effects on lipid metabolism
Hpβ CD	Cyclo Therapeutics	Phase 2b	Small molecule	Cyclodextran derivative to modulate cholesterol transport
ApoE4 LYTAC	Lycia Therapeutics	Pre-clinical	Small molecule	Facilitate degradation of ApoE protein
ApoE4 degrader	Origami Therapeutics	Pre-clinical	Small molecule	Facilitate degradation of ApoE protein
Research program	RAMO	Pre-clinical	Small molecule	ABCA1 agonism
INV-25	Innovimmune	Pre-clinical	Small molecule	LXR selective agonist
Research program	Selonterra	Pre-clinical	Small molecule	Restore *APOE4*-induced gene expression dysfunction and synaptic deficits
CS6253	Artery Therapeutics	Phase 1	Peptide	ABCA1 agonist
CN-105	AnJi pharma/Investigator initiated	Phase 1 (in ICH)	Peptide mimetic	Mimicks ApoE binding to receptors to exert anti-inflammatory activity
LX1001	Lexeo Therapeutics	Phase 1	Gene therapy	ApoE2 augmentation to mitigate effects of endogenous ApoE4
AMT-240	UniQure	Pre-clinical	Gene therapy	Gene silencing of *APOE4* in combination with expressing ApoE2
CLRI-002/CLRI-003 (dCas9)	CLAIRIgene	Pre-clinical	Gene therapy	Gene silencing of *APOE4*
LX1020	Lexeo Therapeutics	Pre-clinical	Gene therapy	Knockdown *APOE4* while expressing *APOE2*
LX1021	Lexeo Therapeutics	Pre-clinical	Gene therapy	Expression of *APOE2* Christchurch to mitigate effects of endogenous ApoE4
RZ-003	Rznomics	Pre-clinical	Gene therapy	AAV delivery of RNA to facilitate trans-splicing of *APOE4* mRNA
Base editing E4 to E2	Editas Medicine	Pre-clinical	Gene therapy	Gene editing to convert *APOE4* to *APOE2*
Research program	GABAeron	Pre-clinical	Cell therapy	Replace GABAergic neurons selectively lost in *APOE4* carriers
Kisbee Disc	Kisbee Therapeutics	Pre-clinical	Lipid disk	Increase ApoE lipidation and function

The list of preclinical programs is not meant to be exhaustive, but rather to illustrate the extent of effort underway and the different modalities being deployed

*AAV* adeno-associated virus, *ASO* antisense oligonucleotide, *CETP* cholesteryl ester transfer protein, *GABA* gamma-aminobutyric acid, *HeFH* Heterozygous familial hypercholesterolemia, *Hpβ CD* hydroxypropyl beta cyclodextrin, *HSPG* heparan sulfate proteoglycan, *ICH* intracerebral hemorrhage, *LXR* liver X receptor, *LYTAC* lysosome-targeting chimeras, *RISC* RNA inducing silencing complex, *siRNA* small interfering RNA

In humans, *APOE* exists as three polymorphic alleles (ε2, ε3 and ε4), which encode protein isoforms that differ from one another only at positions 112 and 158 (Fig. 4). These somewhat modest sequence differences substantially alter the structure and function of ApoE, modulating binding to both lipids and receptors [179, 180]. Relative to the other two allelic forms, *APOε4* confers an increased risk for, and decreased age of, onset of, AD in a gene dose-dependent manner [181]. Indeed, the effect is so pronounced (in certain populations) that some researchers have proposed *APOε4* homozygotes be considered a predetermined form of AD (i.e., Stage 0) [182]. Compared to *APOε3* and *APOε4*, *APOε2* is protective [181].

The greatest driver of recent advances in understanding the role of the *APOE* genotype in AD have come from the identification of rare protective variants, which collectively suggest that ApoE4 mediates a toxic gain of function [183]. Study of the so-called Christchurch (*ch*) variant has been particularly instructive. This variant encodes a single amino acid substitution, R136S, and delayed onset of cognitive impairment by three decades in a woman with an AD-causing mutation in *PSEN1* [79]. Importantly, both PET imaging and subsequent postmortem analysis of this homozygous case indicated very high levels of amyloid pathology, but remarkably limited tau pathology [184].

These and other human data [185], together with recent animal studies [175, 176], indicate that *APOε3*-*ch* protects against the development of tau pathology. Indeed, earlier seminal studies showed that *APOε4* expression exacerbates pathology in tau transgenic mice [170], whereas ablation of *APOE* is protective, and expression of *APOε3* and *APOε2* have intermediate effects [170]. Notably, in humans, loss-of-function mutations in *APOε4* also appear to be protective [177].

ApoE plays a critical role in redistributing cholesterol and other lipids to neurons through binding to cell-surface receptors, including the low-density lipoprotein receptor (LDLR) and LDLR-related protein 1 (LRP1). In addition, ApoE also binds to cell surface heparan sulfate proteoglycans (HSPGs) [186, 187], and HSPGs and LRP1 have been reported to facilitate neuronal uptake of extracellular tau [188, 189]. Interestingly, of the three ApoE isoforms, ApoE4 has the highest affinity for heparin [190, 191], whereas the reduced binding of ApoE3-*ch* to HSPG and LDLR is akin to ApoE2 [79]. Notably, human neurons expressing *APOE4*-*ch* demonstrate reduced tau uptake and p-tau accumulation [175], and treatment of *APOE4* neurons with heparin reduced tau uptake to a level comparable to uptake in *APOE4-ch* and *APOE-KO* neurons. In contrast, when *APOε4-ch* neurons were treated with heparin, there was little or no effect on tau uptake. These results suggest that the *ch* mutation reduced the ApoE4-promoted tau uptake, at least in part, due to its attenuated HSPG binding.

In various human induced pluripotent stem cell-derived brain cells and human brain tissues, ApoE4 has been shown to alter lipid metabolism and increase lipid droplet accumulation, particularly in so-called droplet-accumulating microglia [171, 178, 192]. Single-nucleus RNA sequencing (snRNA-seq) of AD brain tissue revealed that microglia-expressing ACSL1, a lipid droplet associated enzyme, are most abundant in *ε4*/*ε4* AD cases [178]. Moreover, transcriptomic and cellular studies indicate that ASCL1 expressing microglia have impaired phagocytosis and produce high levels of reactive oxygen species and proinflammatory cytokines. In parallel, conditional expression of *APOE* or *APOε4*, in amyloid and tau mouse models, was shown to impair microglial response to amyloid pathology and tau-mediated neurodegeneration [193, 194]; whereas deletion of microglial *APOε4* restored protective microglial responses and astrocyte activation [193]. Thus, in humans and mice, APO*ε4* genotype has been demonstrated to negatively contribute to amyloid and tau pathology by impairing the metabolism and activity of microglia.

In contrast to the other three primary targets considered in previous sections of this review, ApoE has the potential to influence multiple pathogenic pathways in AD, and although ApoE can and does aggregate, the opportunities for drugging ApoE go beyond the strategies discussed for Aβ, tau and ⍺Syn (Fig. 2). There are at least eight different possible intervention points: (1) gene editing, (2) APO*ε2* augmentation, (3) ApoE lowering (including specific forms and fragments), (4) LXR agonism, (5) ATP-binding cassette transporter A1 (ABCA1) upregulation, (6) enhance ApoE lipidation, (7) reduce receptor and/or heparin binding, and (8) modulate lipid metabolism (Table 4).

Gene editing to convert APO*ε4* to APO*ε2,* or APO*ε2ch* has the potential to provide optimal therapeutic benefit, as it should address both the toxic gain and loss of functions associated with APO*ε4* and this approach is being pursued by companies such Editas Medicine (Table 4). However, success will require significant technological advances to ensure the safe and widespread delivery of editing machinery across the brain. Augmentation of APO*ε2* is being tested by Lexeo Therapeutics in a small Phase 1 trial (Table 4) to assess the safety and toxicity of intrathecal administration of AAVrh.10hAPOE2 (LX1001) in *APOE4* homozygous patients with AD Stages 3–4. Interim data indicate that ApoE2 levels never approach more than ~4% of ApoE4 levels [195]. The premise of this approach relies on the apparently protective effects of ApoE2 which is strong in non-Hispanic whites and non-Hispanic blacks, but not other ethnic groups [196]. Beyond the challenges all CNS-directed gene therapies face (i.e., achieving effective, safe, and broad delivery), it is likely that a very high degree of APO*ε2* over-expression will be necessary to overcome the negative effects of ApoE4. The observation that APO*ε2/4* individuals have a greater risk for AD than APO*ε3/3* individuals [181] strongly implies that equal expression of ApoE2 with ApoE4 cannot achieve the neutral effect of *APOε3/3* homozygosity. Similarly, while loss-of-function mutations in APO*ε4* appear to protect against AD in the presence of *APOε2* or *APOε3*, loss-of-function mutations in the presence of *APOε4* do not [177]. Thus, augmentation of ApoE2 and simultaneous lowering of ApoE4 (Lexeo and UniQure, Table 4) has a higher chance of success than augmentation of ApoE2 alone. Even so, it is unclear that providing extracellular ApoE2 will be sufficient to overcome some of the negative effects of ApoE4 which may be mediated within cells.

Potential approaches for lowering ApoE4 are similar to those described for APP, tau and ⍺Syn, namely by targeting transcription, translation and protein degradation, but for ApoE, lowering will be required across several different cell types. Approaches which use vector-encoding agents such as ZFP or artificial micro-RNA will require capsid technology advancements to allow delivery across large regions of the brain and to access neurons, glia and vascular cells. Knock-down of *APOE* mRNA with oligonucleotide (siRNA and ASO) approaches are tractable using current technology and have proved successful when tested in both tau and amyloid mouse models [197–200]. Currently there are at least two pre-clinical stage *APOE* mRNA-targeting programs in development (Table 4). Since delivery of standard oligonucleotides requires intrathecal administration, a focus of these and future programs should be to maximize potency and durability to ensure infrequent dosing regimens. A benefit of intrathecal delivery for *APOE* targeting is the fact that the CNS and peripheral ApoE pools are separate, and specific downregulation in the brain should not impact beneficial effects of ApoE in the periphery. Indeed, it is worth recalling that high levels of ApoE in CSF are associated with increased risk of AD [183], whereas high levels of ApoE in blood appear protective [201, 202].

For patient convenience, it would be desirable to deliver drug by a route other than intrathecal delivery. However, current TfR-based systems should be avoided since these may result in considerable exposure in the liver which is the primary site of peripheral ApoE production. Indeed, it is known that complete loss of ApoE can cause severe, but clinically manageable dyslipidemia [203]. Thus, while rare loss-of-function mutations found in humans indicate that lowering ApoE4 levels in the CNS by at least 50% will have benefit, and that lowering of peripheral levels of ApoE4 to 50% is tolerated [177], careful assessment of the effects on lipid metabolism in each of these pools will nonetheless be required.

Enhancement of ApoE4 degradation with small molecules is appealing, although it too would need to achieve a balance between sufficient lowering in the brain to drive a therapeutic effect, versus minimal lowering in the periphery, to avoid unwanted side effects. Use of molecular glues to selectively degrade ApoE4 and have activity spatially restricted to the brain are particularly appealing. Other means to reduce ApoE akin to those described for tau and ⍺Syn (see above) should also be considered. Alternatives to lowering total levels of ApoE4 is to target those forms of ApoE4 most likely to mediate disease. The two most discussed pathogenic forms are: (1) poorly lipidated and aggregated ApoE, and (2) neuronal fragments of ApoE. Unlipidated aggregated ApoE has been shown to promote a pro-inflammatory response and to seed amyloid plaque formation in mice [204]. Targeting extracellular aggregated ApoE4 has the benefit that it should not engage or affect the physiological function of ApoE, but remove pre-existing plaques and CAA. HAE-4, an antibody (Table 4) which preferentially binds non-lipidated, aggregated forms of ApoE, was shown to reduce existing amyloid pathology in the brain parenchyma and cerebral vessels of 5xFAD mice, and to improve cerebrovascular functions as well as suppress Aβ-mediated tau propagation [205–207]. However, focusing on a single form of ApoE will likely have a more limited effect than knocking down all forms of ApoE4 and there is currently no evidence that such an approach can alter tau-mediated neurodegeneration.

Under physiological conditions, neurons express low levels of ApoE, however there is evidence that expression is upregulated in response to stress and that increased expression of ApoE4 triggers loss of inhibitory inputs and consequent excitotoxicity [208]. Although the mechanisms linking neuronal ApoE4 to pathogenesis are not well understood, it appears that proteolytic cleavage may be important. ApoE4 is more susceptible to cleavage than ApoE3, and in various cell and mouse models C-terminally truncated fragments appear to elicit accumulation of phospho-tau and neurodegeneration [209, 210]. In addition to ApoE expression, it is plausible that inhibition of ApoE cleaving proteases could be of therapeutic value, but the identity of the cleaving activity (or activities) has not yet been elucidated.

Since ApoE4 is known to be poorly lipidated [211, 212], alter lipid metabolism, and cause lipid accumulation, there is growing interest in approaches to increase lipid efflux in glia by agonizing LXR or increasing ABCA1 expression/activity (Table 4). LXR and RXR agonists have been shown to enhance ApoE secretion and lipidation in human astrocytes [213, 214] and the LXR agonist, GW3965 decreased tauopathy, neurodegeneration, inflammation and lipid accumulation in tau transgenic mice [215]. While these pre-clinical data are encouraging, LXR agonism carries serious peripheral liabilities [216, 217] which necessitate LXR agonism be restricted to the CNS. New technologies such as those being developed by Montara [218] could potentially overcome this limitation. In parallel, efforts should continue to identify LXR-independent mechanisms to increase ApoE lipidation [219] (Donovan et al. 2025, in review).

Mimicking the differences between ApoE2/ApoE*ch* and ApoE4 provides a strong rationale for efforts aimed at reducing ApoE4 binding to LDLR and HSPG. Two ApoE peptide mimetics are in clinical trials, albeit for indications other than AD. CN-105 (derived from the receptor binding domain) is in Phase 2 for supratentorial intracerebral hemorrhage, and AEM-28 (which spans the HSPG and lipid binding domains), is an investigator-initiated Phase 1 (Table 4). In addition, an antibody, 7C11, which preferentially binds ApoE4, disrupting heparin-ApoE4 interactions, is in pre-clinical development and was recently shown to reduce tau pathology in the retina of P301S tau transgenic mice and to decrease pTau396 in brains of *APOE4* mice [220].

There is also considerable interest in discovering means to ameliorate ApoE4’s negative effects on cholesterol regulation by promoting expression of cholesterol metabolism-related genes and cholesterol esterification to mirror that of ApoE2 [171, 173, 174]. The local and long-range regulation of *APOE* expression has been neglected, yet the impact of *APOE* genotype on AD risk varies with ancestry [196] and preliminary data suggest this is due to differential regulation of *APOE* [221]. Clearly a deeper understanding of the regulation of *APOE* expression should identify novel targets and mechanism to affect knockdown of ApoE.

### Patient population and biomarkers consideration for APOE/ApoE targeting interventions

Stratification of patients by *APOE* genotype (for which there are existing commercial assays) is already commonplace in AD trials. Notably, in the Phase 3 trials of Lecanemab and Donanemab, *APOE4/4* patients were found to have higher risk for developing ARIA and obtained less clinical benefit than subjects with other *APOE* genotypes. Other E4 carriers derived more benefit, but had an intermediate risk of developing ARIA [8, 9]. Given the high proportion of *APOE4* carriers in the AD population and the strong pathogenic effects of *APOE4*, it is crucial to devise safe therapeutic strategies for this population, particularly E4/E4 subjects [180, 222, 223].

As ApoE4 promotes Aβ-mediated tau pathogenesis, neurodegeneration and microglial functions, ApoE down-regulation through IT delivery of di-siRNA is expected to be most beneficial in early and mild symptomatic (AD stages 3 & 4) *APOE4/4* patients. Targeted removal of (putatively toxic) aggregated ApoE4 with an antibody, is anticipated to: (i) reduce amyloid plaque and CAA, and (ii) improve vascular function, and is expected to be most beneficial in *APOE4/4* patients with AD stages 3 & 4. For *APOE4* heterozygous patients (i.e., *APOE3/4* and *APOE2/4*), it may be more desirable to employ a targeting strategy through small molecule modulation of ApoE-mediated lipid metabolism.

Future trials testing APOE/ApoE targeting agents will likely deploy the advanced tools reviewed in previous sections of this article, i.e., assessment of amyloid by PET, CSF tau, plasma pTau217, and perhaps use of tau PET and/or future fluid markers of tau pathology (e.g., MTBR243). However, ApoE specific target engagement biomarkers will be required. For ApoE lowering programs ApoE will be measured in CSF and blood. Whereas, for programs targeting aggregated ApoE, specific clinical grade assays will need to be developed. Methods to monitor change in BBB and vascular integrity will also be required.

## Other potential targets

While this review focuses on the 4 targets for which we believe are the most tractable and best supported by human data, a mind-boggling array of other targets, biologies, non-pharmacological interventions, and lifestyle approaches are being considered for the management and treatment of AD [224]. As of January 25, 2025 there were 138 agents in 182 clinical trials for AD [225]. This pipeline includes 48 trials in Phase 3, 86 trials in Phase 2, and 48 trials in Phase 1. Disease-modifying therapies represent 82.4% of the total number of agents in trials; with symptomatic cognitive enhancing treatments and drugs for neuropsychiatric symptoms comprising the remainder. Thirty three percent of the candidate agents in the pipeline are repurposed drugs approved for other indications.

Besides the 4 core targets which we deem the most tractable, other efforts can be considered in 7 broad classes: (1) neuroimmunology, (2) neurotransmitter, (3) vasculature (4) metabolic, (5) neurogenesis, (6) epigenetic regulators, and (7) growth factors and hormones [226]. Of the disease modifying approaches, modulation of inflammation and immunity is the most intensely investigated [225].

Proliferation and activation of microglia is a long-established feature of AD [227], but interest in the role of microglia in AD has increased in the past decade because of a series of genetic discoveries. Groundbreaking Genome Wide Association Studies (GWAS) identified both rare coding mutations as well as common genetic variants enriched in microglia [228]. Rare coding variants (e.g., R47H and R62H) in TREM2 significantly increase risk of developing AD [229, 230]. In the brain TREM2 is exclusively expressed on microglia, and homozygous mutations in TREM2 or its signaling partner DAP12 cause an early onset dementia known as Nasu-Hakola disease [231]. In parallel, GWAS studies identified common SNPs at loci with microglial-specific expression patterns (e.g., CD33, ABCA7, and MS4A) [228, 232]. While, almost 80% of AD genetic loci are in genes expressed in microglia, most are also expressed by peripheral immune cells, and it is not clear what effect the peripheral immune system has on AD, or how it should be modulated therapeutically. Moreover, immune-associated GWAS hits appear to act at the level of increasing risk, and not necessarily altering disease progression. Consequently, while targeting microglia and neuroinflammation is of considerable interest, such approaches are filled with risk.

Under certain conditions inflammation can be beneficial, whereas under other conditions it can be harmful. For instance, anti-inflammatory approaches may prove detrimental during the “amyloid phase” of the disease, but beneficial in the later tau-driven phase. In terms of the stages of intervention currently feasible in AD (i.e., early symptomatic disease) different parts of the brain may require different types of microglia or immunological modulation. Primary or secondary prevention may be the optimal time to modulate microglia and other aspects of the immune system, particularly when there is genetic evidence for modulation of disease risk. Nonetheless, it is important to remember that microglia do not exist in a binary state, i.e., good vs. bad, but rather as a continuum of states [233]. Thus, knowing how to modulate microglia and being able to measure the change in vivo is challenging.

## Combination therapy

AD is a multifactorial disease, in which many changes arise simultaneously or close in time, and the precise disease process is unlikely to be the same for all patients. This is particularly true in older populations wherein AD often occurs in the setting of mixed pathologies and on the backdrop of a range of co-morbidities [234]. Individual susceptibilities (including genetic and environmental factors) are also expected to influence the form and course of the disease. Moreover, AD pathology is not uniform across the brain, but shows a rather specific spatiotemporal evolution. This means that at different stages of disease, different molecular pathology processes are occurring simultaneously in different regions of the same brain. Beyond primary prevention, for which anti-amyloid monotherapy may be sufficient, it has long been anticipated that the effective treatment for the symptomatic phases of AD will require the use of 2 or more disease-modifying drugs [235]. Since several recent reviews have been dedicated to the topic of combination therapy in AD [236–238], here we briefly discuss: (i) some basic principles, (ii) examples of ongoing or recently terminated programs, and (iii) approaches most likely to be tested in the near future.

Typically, combination therapies are developed by one of 2 routes – by “add-on” strategies in which a new molecular entity (NME) is tested on top of the current standard of care, or by testing two or more NMEs simultaneously. The latter requires that there is a sound biological rationale to justify combining the two agents and when used together the benefit is superior to that produced by the individual components (i.e., demonstrated proof of synergy). The trial design to prove superiority generally requires 4 arms—comparing A + B vs. A vs. B vs. placebo. If endpoints such as CDR-SB are used to measure clinical efficacy the size of such 4 arm studies would be prohibitive. Thus, add-on studies are the most tractable means to advance combination therapy in AD. Add-on approaches can be tested either by directly combining an approved drug with an NME, or by testing an approved drug either before or after an NME (i.e., in sequence). While such trials do not ensure a label for a ‘combination’, neither do they need to demonstrate superiority and consequently fewer arms are required and the sample sizes are smaller. A drawback of testing agents in sequence is that the study may be longer in duration.

Combination studies are not new in AD, indeed several symptomatic and lifestyle treatments have been combined and tested, and the FDA approved the combination of memantine and donepezil (Namzaric) for moderate to severe AD in 2014 [239]. Moreover, all recent phase 3 trials of disease modifying therapies in AD Stage 3 and 4 have been conducted on stable standard of care symptomatic treatments (e.g., acetylcholine esterase inhibitors, and/or memantine). However, it was only in 2017 that the first combination trial of potential disease modifying therapies was initiated. The 3-armed Phase 2 combination trial involving what was then 2 NMEs: the anti-Aβ antibody, donanemab, and the BACEi, LY3202626. While the principal study objective was to test safety and tolerability, it was also intended to allow comparison of donanemab alone versus donanemab plus LY3202626. The combination of donanemab and LY3202626 was intended to simultaneously reduce Aβ production and remove amyloid plaques (Fig. 2, points 1 and 5) and thus to remove and/or prevent the production of all potentially noxious forms of Aβ. Unfortunately, due to safety concerns about the use of high dose BACEi (see Sect. 2, above), the donanemab + LY3202626 arm was discontinued. While the future use of BACEi’s are uncertain, the prospect of combining a GSM with lecanemab or donanemab offer considerable promise. This could be done by (1) simultaneously administering GSM with an approved anti-Aβ immunotherapy, or (2) by first reducing amyloid below 24 centiloids and then using a GSM for maintenance. In the case of simultaneous administration, one might anticipate faster clearance of plaques and the potential use of lower doses of antibody and hence reduced incidence of ARIA, whereas the sequential approach would enable a low-cost maintenance regimen and reduce patient burden. The most notably ongoing combination study is testing 6 month treatment with lecanemab followed by treatment with the anti-tau antibody, E2814; or 12 month treatment with E2814 followed by lecanemab [240]. This trial is likely a harbinger of many different studies that will test combination of anti-Aβ immunotherapy with a tau targeting therapy. This is both biological rational and pragmatic, rational because this approach targets the 2 main pathologies of AD and simultaneously addressing both is expected to have more benefit across a broad range of disease stages (Fig. 1), and pragmatic because Aβ immunotherapy is likely to become standard of care in certain geographies such that the testing of tau targeting agents may need to be done as add-on’s. Depending on the outcomes of ongoing responder-non-responder analyses for approved anti-Aβ immunotherapies add-on therapies targeting co-pathologies such as ⍺Syn, vascular dysfunction, lipid dyshomeostasis and inflammation may also move forward.

## Conclusions

It is evident from the material reviewed that there is already considerable activity on the four targets we recommend for prioritization. Yet, there remains a need for technical innovations to ensure development of truly efficacious therapies and for the application of CRISPR screens (and similar technologies) to identify new nodes to regulate APP, tau, ⍺Syn and ApoE. BBB shuttles to enable widespread distribution across the brain are a welcome addition to the armamentarium of drug developers, but how and why they are used differs for the modalities to which they are applied and the targets for which they are intended. For Aβ immunotherapy, BBB shuttles have the potential to significantly increase brain exposure while reducing risk for ARIA. Whereas, for oligonucleotides the primary utility of BBB shuttles in AD is to avoid intrathecal delivery. While the latter is highly desired by patients and providers, intravenous delivery also brings the potential of unwanted effects in the periphery. This is not an issue for targets such as tau, but knockdown of *APP* and *APOE* in the periphery could be problematic. Consequently, other delivery mechanisms such as CNS-addressed lipid nanoparticles and exosomes are worthy of exploration. Similarly, squaring the circle of deploying oral small molecules to enable patient friendly efficient transfer into the CNS while mitigating peripheral liabilities will require target specific innovations.

Many exciting new modalities such as degraders and molecular glues are starting to have success in non-CNS indications and should be robustly tested for AD and other diseases of the brain. Similarly, gene and cell therapy offer great promise for the delivery of vectorized antibodies and the editing and silencing of targets, but to realize these “one and done” treatments of the future will require significant investment in technology to enable widespread delivery in the brain.

A lot of hard work will be required to deliver the next wave of AD disease modifying therapies, but the future is bright. We have the right targets, and emerging technologies are here or near, such that we are confident that persistent effort will ultimately pay dividends for sponsors and patients. The lofty goal of reaching as many patients as possible, as early in the disease process as possible, will only be realizable by the development of low cost, low burden treatments. However, we are mindful that this will likely be an incremental process, and that less convenient more restricted proof-of-concept treatments may be necessary intermediates on the path to ultimate success.

## Data Availability

All data cited in the text is already in the public domain.

## Footnotes

1 While it is widely assumed that reduction in amyloid and tau PET signals seen with anti-Aβ immunotherapy is largely due to removal of amyloid plaques or slowing of tangle accumulation only limited postmortem studies quantifying plaques in treated patients have been conducted.
